# Evaluating the Unmet Needs of Older Adults to Promote Functional Recovery After the ICU: The LANTERN Study

**DOI:** 10.1093/geroni/igaf122.2189

**Published:** 2025-12-31

**Authors:** Allison Cho, Nicole Bouranis, Janet Truebig, Nalani Walker, Amy Shelton, Andrea Benjamin, Jason Falvey, Lauren Ferrante

**Affiliations:** Yale School of Medicine, New Haven, Connecticut, United States; Yale School of Medicine, New Haven, Connecticut, United States; Yale School of Medicine, New Haven, Connecticut, United States; Yale School of Medicine, New Haven, Connecticut, United States; Yale School of Medicine, New Haven, Connecticut, United States; Yale School of Medicine, New Haven, Connecticut, United States; University of Maryland School of Medicine, Baltimore, Maryland, United States; Yale School of Medicine, New Haven, Connecticut, United States

## Abstract

Each year, nearly two million older adults survive a critical illness in the U.S., with half experiencing increased functional disability thereafter. No studies have focused on improving post-ICU functional recovery among older adults. Preliminary data suggests unmet needs within four domains (home environment, skilled rehabilitation, hearing/vision, caregiving) hinder functional recovery. LANTERN is a longitudinal study enrolling older ICU survivors. The objectives are to (1) identify unmet needs in these four domains 2-4 weeks after the patient returns home and evaluate associations with disability, rehospitalizations, and mortality over the subsequent six months, and (2) determine facilitators and barriers to addressing unmet needs. At enrollment, the home is thoroughly examined for barriers to functional recovery, such as needing grab bars in the shower. Participants are asked about needs in skilled rehabilitation and caregiving services. They undergo subjective and objective hearing/vision assessments, and a comprehensive geriatric assessment to inform covariates. Six monthly follow-up phone interviews ascertain disability in 15 functional activities, rehospitalizations, vital status, and changes to the domains of unmet needs. Data extraction from the electronic medical record (EMR) supplement the interviews. After the six-month visit, participants and caregivers are interviewed to determine facilitators and barriers to addressing unmet needs. To date, we have screened 1462 patients: 770 were eligible, 238 consented in-hospital, and 92 enrolled. Of enrolled participants, the mean age (SD) was 74.8 (7.1). This study elucidates whether unmet needs among older ICU survivors are associated with subsequent disability burden, rehospitalizations, and mortality, informing future interventions to support functional recovery.

